# Risk prediction of 90-day complications in implant-based breast reconstruction –does previous post-bariatric massive weight loss add to the risk? A population-based study

**DOI:** 10.1016/j.jpra.2026.07.041

**Published:** 2026-08-08

**Authors:** Emma Hansson, Ying Li, Anna Grimby-Ekman, Johan Ottosson, Anna Paganini

**Affiliations:** a Department of Plastic Surgery, Institute of Clinical Sciences, The Sahlgrenska Academy, University of Gothenburg, Gothenburg, Sweden; b Department of Plastic Surgery, Sahlgrenska University Hospital, Gothenburg, Region Västra Götaland, Sweden; c School of Public Health and Community Medicine, Institute of Medicine, Sahlgrenska Academy, University of Gothenburg, Gothenburg, Sweden; d Department of Surgery, Faculty of Medicine and Health, Örebro University, Örebro, Sweden

**Keywords:** Massive weight loss, Bariatric surgery, Breast reconstruction, Risk prediction, Breast implant

## Abstract

**Introduction:**

Risk stratification is essential when selecting the timing and technique of breast reconstruction. While several prediction models exist, none have incorporated prior massive weight loss (MWL), despite a growing population of such patients. This study aimed to develop cumulative risk prediction models for complications after implant-based breast reconstruction, explicitly including MWL as a candidate predictor.

**Methods:**

This nationwide, population-based case–control study used prospectively collected Swedish registry data. Patients with prior bariatric surgery who underwent immediate or delayed post-mastectomy implant-based breast reconstruction between 2007 and 2022 were identified and matched to at least 3 controls by age and body mass index. Candidate predictors were selected based on prior literature. Group least absolute shrinkage and selection operator (LASSO) regression with bootstrap resampling was used to develop prediction models, which were visualized as nomograms.

**Results:**

A total of 245 immediate and 602 delayed breast reconstructions were included; 11% and 6%, respectively, had a history of MWL. Complication rates were 16.3% for immediate and 14.5% for delayed reconstruction. Prior MWL was consistently selected as an independent predictor of complications in both immediate and delayed reconstruction models and of re-admission after delayed reconstruction. Other predictors included age, body mass index, radiotherapy, comorbidity-related medications, and antibiotic use.

**Conclusion:**

Prior massive weight loss following bariatric surgery is an independent risk factor for complications after both immediate and delayed implant-based breast reconstruction. Incorporating MWL into cumulative risk prediction models improves individualized risk assessment and supports informed, shared decision-making in reconstructive breast surgery.

## Introduction

Breast reconstruction is an integral part of breast cancer treatment, but as an elective procedure, the risk of complications must be carefully considered when timing and technique are chosen. There are well-known risk factors for complications in breast reconstruction, including patient-related factors such as a high body mass index (BMI), smoking, comorbidity, and age,^1^^,^ ^2^ and treatment-related factors such as radiotherapy^1^ and the timing of reconstruction.^3^ Previous studies often focus on calculating risks for individual factors rather than on cumulative risks in patients with multiple risk factors.^1^ To our knowledge, six studies have focused on cumulative risk prediction in breast reconstruction.^4^, ^5^, ^6^, ^7^, ^8^, ^9^ Details on the studies and risk factors are provided in Supplementary Table 1. None of the studies incorporated post-bariatric status/massive weight loss (MWL) as a candidate predictor in their model.

Bariatric surgery is widely performed worldwide, with approximately 6.5 million procedures carried out between 2008 and 2018.^10^ In addition, medically induced MWL has increased with the widespread use of Glucagon-Like peptide-1 (GLP-1) receptor agonists, such as Semaglutide, sometimes in combination with bariatric surgery.^11^ Given that breast cancer is the most frequently diagnosed cancer among women, a substantial number of individuals who have undergone MWL will later develop breast cancer.^12^ Furthermore, bariatric surgery has been proposed as a preparatory or bridging intervention to reduce risks in patients with obesity who are seeking breast reconstruction.^13^ Consequently, reconstructive surgeons are expected to encounter a growing population of patients who have experienced MWL and require breast reconstruction. This necessitates knowledge of the effect of MWL on the risk of complications in breast reconstruction.

MWL can alter tissue quality, potentially weaken mechanical properties, and increase the risk of surgical complications.^14^ In addition, individuals who have undergone bariatric surgery may develop nutritional deficiencies that negatively affect tissue repair and wound healing.^15^, ^16^, ^17^ Studies evaluating body contouring procedures after MWL have consistently shown a significantly higher complication risk in patients with prior MWL compared with those without.^18^ Specifically for breast reconstruction after MWL, a recent systematic review^19^identified only 15 previous studies. Of these, three were case–control studies, including 6–39 cases and 18–1012 control subjects, while the remaining 12 consisted of case reports or case series. Only one study specifically addressed implant-based breast reconstruction in patients with MWL, reporting a case series of 28 implant-based reconstructions performed in 20 MWL patients.^20^ Notably, none of the studies included in the review evaluated MWL as an independent risk factor for postoperative complications.

This study aimed to develop a prediction model for the risk of complications in implant-based breast reconstruction after therapeutic mastectomy, incorporating previous massive weight loss/bariatric surgery as a candidate predictor. We hypothesized that massive weight loss is a risk factor that increases the cumulative risk of complications.

## Methods

### Protocol and sample

This investigation employed a nationwide, population-based case–control design using prospectively collected data from national registries. It is part of *the Breast Reconstruction After Bariatric Surgery study* (ClinicalTrials.gov identifier NCT07059104) and was conducted in accordance with the RECORD extension of the STROBE reporting guidelines.^21^ Comprehensive descriptions of the registries used, and the criteria for identifying cases and controls have been published previously.^22^ The protocol includes one benign arm with patients and controls undergoing breast augmentation and one malignant arm with patients and controls undergoing post-mastectomy breast reconstruction. This study focuses on risk factors for complications in the malignant arm. Only patients who had a therapeutic mastectomy were included in the present study.

The study cohort included all individuals who underwent bariatric surgery between 2007 and 2022 and were registered in the Scandinavian Obesity Surgery Registry (SOReg), who subsequently underwent post-mastectomy immediate breast reconstruction recorded in the Swedish Breast Cancer Registry (NKBC), or post-mastectomy delayed breast reconstruction recorded in the Swedish Breast Implant Registry (BRIMP). Controls from NKBC and BRIMP were matched to patients by age and body mass index (BMI) at the time of breast reconstruction. All mastectomies were therapeutic, and any risk-reducing mastectomies were excluded. The matching was conducted by statisticians at the Centres of Registers Västra Götaland and Stockholm, with a minimum of three controls assigned to each patient.

### Setting

In Sweden, bariatric surgery is typically offered to individuals with a body mass index (BMI) of 35 or above, as well as to those with a BMI between 30 and 35 who have significant obesity-related comorbidities, particularly when non-surgical weight loss methods have proven unsuccessful. Each year, around 5000 such procedures are carried out nationwide.^23^ According to the Swedish guidelines, breast reconstruction is offered to women who have a BMI of 30 or below, do not have metastasised breast cancer, and who refrain from smoking for a minimum of six weeks both before and after the surgery. If the patient has had a bariatric operation, she must be weight stable for at least 6 months at the time of any plastic surgical procedure, such as breast reconstruction. All aspects of breast reconstruction after mastectomy are provided within the publicly funded healthcare system. According to Swedish national guidelines, patients have to have a BMI under 30 to undergo breast reconstruction.

### Ethics

The study received ethical approval from the Swedish Ethical Review Authority (reference number 2024–01,494-01). It was conducted in accordance with the Declaration of Helsinki as incorporated into Swedish legislation through the Ethics Review Act (SFS 2003:460). All data handling complied with the General Data Protection Regulation (GDPR) and the study’s approved data management plan. For registry-based research in Sweden, individual informed consent is not required because the data are pseudonymized, and consent for research use is obtained at the time individuals are enrolled in the national registries.

### Data sources and variables

Data on bariatric operations were retrieved from SOReg, and data on implant-based breast reconstruction from BRIMP and NKBC. Additional data were collected for the 3 months before and 3 months after breast reconstruction from the National Patient Registry (PAR) and the National Prescribed Drug Register (PDR).

*SOReg* has recorded information on nearly all patients undergoing bariatric surgery in Sweden since 2008, capturing approximately 99% of cases. The register documents surgical procedures, patient demographics, weight-loss outcomes, and postoperative complications.^23^ NKBC has been operational since 2008 and collects nationwide data. It includes 99.9% of all patients who undergo breast cancer surgery in the country, with concordance for the surgical variables analysed in this study ranging from 96% to 99%.^24^ The completeness of NKBC has been evaluated through linkage with the Swedish Cancer Register (SCR), and its validity has been confirmed by re-abstraction of medical records.^24^ BRIMP was initiated in 2011 and achieved nationwide coverage by 2014. As of 2021, the registry contained information on over 50,000 breast implants. Although participation by surgeons is voluntary, approximately 85% of private practitioners contribute data. The registry provides detailed information on surgical methods, implant specifications, and follow-up and revision procedures.^25^ PAR includes data on inpatient care dating back to 1964, with nationwide completeness achieved from 1987 onward. In addition, it has recorded all specialized outpatient visits since 2011 and covers nearly all public healthcare services in Sweden.^26^ The register does not include information from primary care. Diagnoses in PAR are coded using the International Statistical Classification of Diseases and Related Health Problems, Tenth Revision (ICD-10). *PDR* contains records of medications prescribed in Sweden by physicians in both public and private practice and has high completeness due to compulsory reporting. Medications administered during hospital stays are not included, as they are not prescribed to individual patients.^27^ Drugs in the PDR are classified according to the Anatomical Therapeutic Chemical (ATC) system.

Follow-up was calculated from the date of data retrieval (27.03.2025) or from the date of death, if the patient was deceased. MWL was defined as a patient who had undergone bariatric surgery according to SOReg. Surgical variables and complications were defined as in SOReg, NKBC, and BRIMP. As only specialist care is registered in PAR, the use of a collection of prescriptions for the following drugs three months before the breast reconstruction was used as an indicator of comorbidity. The following ACT codes were collected in PDR: anticoagulants (B01), diabetes medications (A10), blood clotting drugs (B02), corticosteroids (H02), immunosuppressants (L04), and anti-inflammatory and antirheumatic medications (M01). Substitution drugs were defined as the collection of prescriptions for vitamins and minerals (A11-A12) and/or anemia treatment (B03). Complications were collected from PAR. Any complication was defined as the occurrence of any of the following ICD-10 codes during the first 90 days after the breast reconstruction: T81.3 (rupture of surgical wound), T81.4 (wound healing complication), T81.0 (hematoma after surgery), HAD 50 (implant loss), A41 (septicemia), I80-I80.9, I 26, I81 (deep vein thrombosis/pulmonary embolism), I21 (myocardial infarction), and I61–64 (stroke). Re-admission was defined as any inpatient admission to specialist health care within 90 days after the breast reconstruction, independent of ICD code. Candidate predictors for complications were identified based on previous literature on factors associated with complications^1^^,^ ^2^^,^ 4, 5, 6, 7, 8, 9 and chosen based on available variables in the registries used. All variables listed in Table 1/Supplementary Table 2 were considered for analysis.

### Statistics

Categorical variables are presented as frequencies and percentages, whereas continuous variables are summarised by medians with interquartile ranges (IQR). Patients were classified into two groups: those who underwent a revision and those who did not. Separate analyses were performed for immediate and delayed breast reconstruction, as data were retrieved from two different registries, and the available variables therefore differed slightly for the two groups (Table 1). Comparisons of continuous variables between groups were performed using the Wilcoxon rank-sum test, while categorical variables were compared using either Fisher’s exact test or Pearson’s Chi-squared test. Cases with missing data for one or more candidate predictors were excluded from the analyses.

**Table 1:** Candidate predictors in patients who had and did not have a complication, and differences between the groups.

Candidate predictors for any complication	Immediate breast reconstruction			Delayed breast reconstruction		
	Patients who had no complication N = 250^1^	Patients who had a complication N = 40*^1^*	p-value*^2^*	Patients who had no complications N = 515*^1^*	Patients who had a complication N = 87*^1^*	p-value*^2^*
**Characteristics of participants**						
Postbariatric/MWL			**<0.001**			0.078
No	190 (93%)	28 (70%)		487 (95%)	78 (90%)	
Yes	15 (7.3%)	12 (30%)		28 (5.4%)	9 (10%)	
Missing	0	1		0	0	
BMI at breast reconstruction	Could not be analysed due to missing data			25.5 (23.6, 27.9)	26.3 (24.4, 28.4)	**0.015**
Missing				7	4	
Percentage of BMI lost	Could not be analysed due to missing data			Could not be analysed due to missing data		
Age at breast reconstruction (years)	47 (42,52)	47 (44, 57)	0.4	51 (44, 60)	51 (47, 59)	0.7
Missing	0	0		4	2	
Smoking	Could not be analysed due to missing data			Not available in the registry		
Post-operative complications the first 6 weeks after the bariatric operation^2^	Could not be analysed due to missing data			Could not be analysed due to missing data		
No						
Yes						
**Comorbidity expressed as medication (ACT-codes) taken 3 months before the breast reconstruction**						
Diabetes medication (A10)			>0.9			0.2
No	201 (98%)	40 (100%)		502 (97%)	87 (100%)	
Yes	4 (2.0%)	0 (0%)		13 (2.5%)	0 (0%)	
Vitamins and minerals (A11-A12)^3^			0.063			0.11
No	190 (93%)	33 (83%)		457 (89%)	72 (83%)	
Yes	15 (7.3%)	7 (18%)		58 (11%)	15 (17%)	
Cardiac medications (C01)			0.3			0.3
No	204 (100%)	39 (98%)		204 (100%)	39 (98%)	
Yes	1 (0.5%)	1 (2.5%)		1 (0.5%)	1 (2.5%)	
Anticoagulants (B01)			0.2			0.4
No	198 (97%)	37 (93%)		475 (92%)	78 (90%)	
Yes	7 (3.4%)	3 (7.5%)		40 (7.8%)	9 (10%)	
Corticosteroids (H02)			**<0.001**			0.7
No	184 (90%)	27 (68%)		473 (92%)	81 (93%)	
Yes	21 (10%)	13 (33%)		42 (8.2%)	6 (6.9%)	
Anti-inflammatory and antirheumatic medications (M01)			0.3			0.076
No	195 (95%)	36 (90%)		474 (92%)	75 (86%)	
Yes	10 (4.9%)	4 (10%)		41 (8.0%)	12 (14%)	
Neuroleptics, sedatives and sleeping medications (N05)			0.2			>0.9
No	188 (92%)	34 (85%)		512 (99%)	87 (100%)	
Yes	17 (8.3%)	6 (15%)		3 (0.6%)	0 (0%)	
Asthma/COPD medications (R03)			>0.9			**0.005**
No	196 (96%)	39 (98%)		496 (96%)	77 (89%)	
Yes	9 (4.4%)	1 (2.5%)		19 (3.7%)	10 (11%)	
Blood clotting medications (B02)			>0.9			0.6
No	204 (100%)	40 (100%)		509 (99%)	87 (100%)	
Yes	1 (0.5%)	0 (0%)		6 (1.2%)	0 (0%)	
**Oncological factors**						
Invasive cancer			0.10	Could not be analysed due to missing data		
No	123 (64%)	26 (79%)				
Yes	69 (36%)	7 (21%)				
Missing	13	7				
Neoadjuvant chemotherapy			**0.012**	NA		
No	180 (88%)	29 (73%)				
Yes	25 (12%)	11 (28%)				
Radiotherapy after the reconstruction			**0.053**	NA		
No	97 (62%)	14 (44%)				
Yes	59 (38%)	18 (56%)				
Missing	49	8				
Radiotherapy before the reconstruction	Data not available in the registry					0.2
No				387 (81%)	61 (74%)	
Yes				92 (19%)	21 (26%)	
Missing				36	5	
Number of tumour operations			0.4	NA		
0	162 (79%)	28 (70%)				
1	33 (16%)	10 (25%)				
≥2	10 (4.9%)	2 (5.0%)				
≥2	10 (4.9%)	2 (5.0%)				
Axillary surgery			>0.9	Not available in the registry		
No	14 (6.8%)	2 (5.0%)				
Yes	191 (93%)	38 (95%)				
**Factors related to breast reconstruction**						
Contralateral surgery at the same time as the reconstruction			0.3	Not available in the registry		
No	182 (89%)	33 (83%)				
Yes	23 (11%)	7 (18%)				
Usage of acellular dermal matrix or mesh			0.5			0.5
No	158 (77%)	29 (73%)		500 (97%)	83 (95%)	
Yes	47 (23%)	11 (28%)		15 (2.9%)	4 (4.6%)	
Prepectoral placement of implant			0.4	Classified in another way in the registry		
No	174 (85%)	32 (80%)				
Yes	31 (15%)	8 (20%)				
Tissue plane	Classified in another way in the registry					**0.027**
Submuscular				317 (66%)	41 (51%)	
Pre-pectoral				77 (16%)	20 (25%)	
Subfascial				10 (2.1%)	0 (0%)	
Dual plane				78 (16%)	19 (24%)	
Missing				33	7	
Type of implant	Could not be analysed due to missing data					0.3
Implant				299 (59%)	45 (53%)	
Permanent tissue expander				212 (41%)	40 (47%)	
Missing				4	2	
Implant volume	Could not be analysed due to missing data			Could not be analysed due to missing data		
Implant shape	Data not available in the registry					0.8
Round^4^				64 (12%)	10 (11%)	
Anatomical				451 (88%)	77 (89%)	
Missing						
Implant surface	Data not available in the registry					0.7
Smooth/nano textured				13 (2.5%)	1 (1.1%)	
Micro/macro textured				502 (97%)	86 (99%)	
Polyurethan				0	0	
Incision	Data not available in the registry					0.7
Mastectomy scar				305 (65%)	47 (63%)	
Other				162 (35%)	28 (37%)	
Missing				48	12	
Lipofilling^5^	Data not available in the registry					>0.9
Yes				506 (98%)	86 (99%)	
No				9 (1.7%)	1 (1.1%)	
Aesthetic re-operation	Data not available in the registry					0.6
No				445 (86%)	77 (89%)	
Yes				70 (14%)	10 (11%)	
Perioperative antibiotics (at induction)	Could not be analysed due to missing data					**0.002**
No				63 (13%)	1 (1.3%)	
Yes				411 (87%)	78 (99%)	
Missing				41	8	
Intraoperative antibiotics (rinse)	Data not available in the registry					0.7
No				438 (97%)	76 (99%)	
Yes				13 (2.9%)	1 (1.3%)	
Missing				64	10	
Postoperative antibiotics	Data not available in the registry					0.2
No				256 (56%)	37 (47%)	
Yes				205 (44%)	41 (53%)	
Missing				54	9	

1 Median (Q1, Q3); n (%).

2 Wilcoxon rank sum test; Fisher's exact test; NA; Pearson's Chi-squared test.

3 Variables are defined in the method section.

4 Ergonomic implants are registered as ‘round’ in BRIMP (https://registercentrum.blob.core.windows.net/brimp/r/Br-stimplantatregistret-Prim-roperation-2024–05–17-tvALbnphv.pdf).

5 Lipofilling is only reported in BRIMP, and thus figures for lipofilling performed in conjunction with delayed breast reconstruction are reported. BMI Body mass index. COPD chronic obstructive pulmonary disease. MWL massive weight loss.

To identify the most influential predictors, the grouped least absolute shrinkage and selection operator (group LASSO) method was applied, with model selection guided by cross-validation. To assess the stability of variable selection, bootstrap resampling with 1000 iterations was performed. In each iteration, a new dataset was generated by sampling with replacement from the original data, the variable selection procedure was repeated, and the selected predictors were recorded. This allowed calculation of the selection frequency for each variable across all iterations; for instance, a variable selected in 50% of the iterations indicated moderate stability. To determine an optimal inclusion threshold, models based on predictors selected in over 60% of iterations were compared with those selected in over 50% using the likelihood ratio test. Based on this comparison, consistently selected predictors were retained as candidate variables for the final model. To address multicollinearity among these candidates, group LASSO penalization was reapplied to identify the most influential predictors. Additionally, because separation was observed for some variables, coefficients were estimated using a profile penalized log-likelihood approach. The final models are then established, and a nomogram was subsequently developed to estimate the risk of re-operation or revision.

## Results

The study included 245 immediate implant-based breast reconstructions (IBR), of which 27 (11%) had experienced MWL, and 602 delayed implant-based breast reconstructions (DBR), of which 37 (6%) had experienced MWL. The complication frequencies were 16.3 per cent in the immediate group and 14.5 per cent in the delayed group, and the re-admission rates were 26.9 per cent and 8.8 per cent, respectively, during the 90 days following breast reconstruction (Supplementary Table 3). The median age was 47 years (range 27–71) in the immediate group and 52 years (range 21–79) in the delayed group, and the median duration of follow-up was 87 months (5–218 months) in the immediate group and 55 months (4–145 months) in the delayed group. None of the patients had a bilateral mastectomy and breast reconstruction. Demographic and surgical data on the MWL patients included in the study are given in Table 2. Median weight loss was 30 kg (range 5–72 kg). Median BMI at the breast operation was 26 (range 22–33) among the MWl patients and 26 (15–40) among controls. Very few patients had a BMI over 30.

**Table 2:** Demography of post-bariatric patients that had a breast reconstruction.

Post-bariatric patients that had a breast reconstruction		61 patients (64 breasts)	Missing data
**Age at bariatric operation,** years Mean (SD), Median (min-max)		44 (7.9) 43(28–63)	1
**BMI at bariatric operation**, kg/m^2^ Mean (SD), Median (min-max)		39 (4.1) 38 (31–51)	2
**Weight loss at breast operation**, kg Mean (SD), Median (min-max)		33 (14.8) 30 (5–72)	32
**Percentage of BMI loss** (kg/m^2^) Mean (SD), Median (min-max)		12 (5.9) 11 (4–37)	32
**Time between bariatric operation and breast operation** (years) Mean (SD), Median (min-max)		4.9 (3.7) 3.7 (0.6–14.2)	1
**Type of bariatric surgery** (index operation) n, %	Gastric bypass (GBP)	44 (72%)	0
	Gastric banding (GB)	0	
	Biliopancreatic diversion according to Scopinaro (SCOP)	0	
	Sleeve gastrectomy (SG)	17 (28%)	
	Other method	0	
**Previous bariatric procedures**			
No		60	1
Yes		0	
**Complications during the first 6 weeks after bariatric operation**			
No		54	2
Yes		5	
**Readmission after the bariatric operation**			
No		57	3
Yes		1	

In the IBR group, more patients who later had a complication had experienced MWL (*p* < 0.001), were on corticosteroids (*p* < 0.001), had neoadjuvant chemotherapy (*p* = 0.012), and had received radiotherapy to the reconstructed breast (*p* = 0.052) compared to those who did not have a complication (Table 1). In the DBR group, patients who later had a complication had a higher BMI (*p* = 0.015), were more likely to be on asthma/chronic obstructive pulmonary disease medications (*p* = 0.005), had a pre-pectoral or dual plane covered implant (*p* = 0.027), and received more perioperative antibiotics (*p* = 0.002) than patients who did not have a complication (Table 1). Among patients who were re-admitted within the first 90 days following breast reconstruction, more patients in the IBR group had several tumour operations (<0.001), and more patients in the DBR group had a higher BMI (*p* = 0.004), were on blood clotting drugs (*p* = 0.010), and received more perioperative antibiotics (*p* = 0.031) (Supplementary Table 2).

**Fig. 1:**
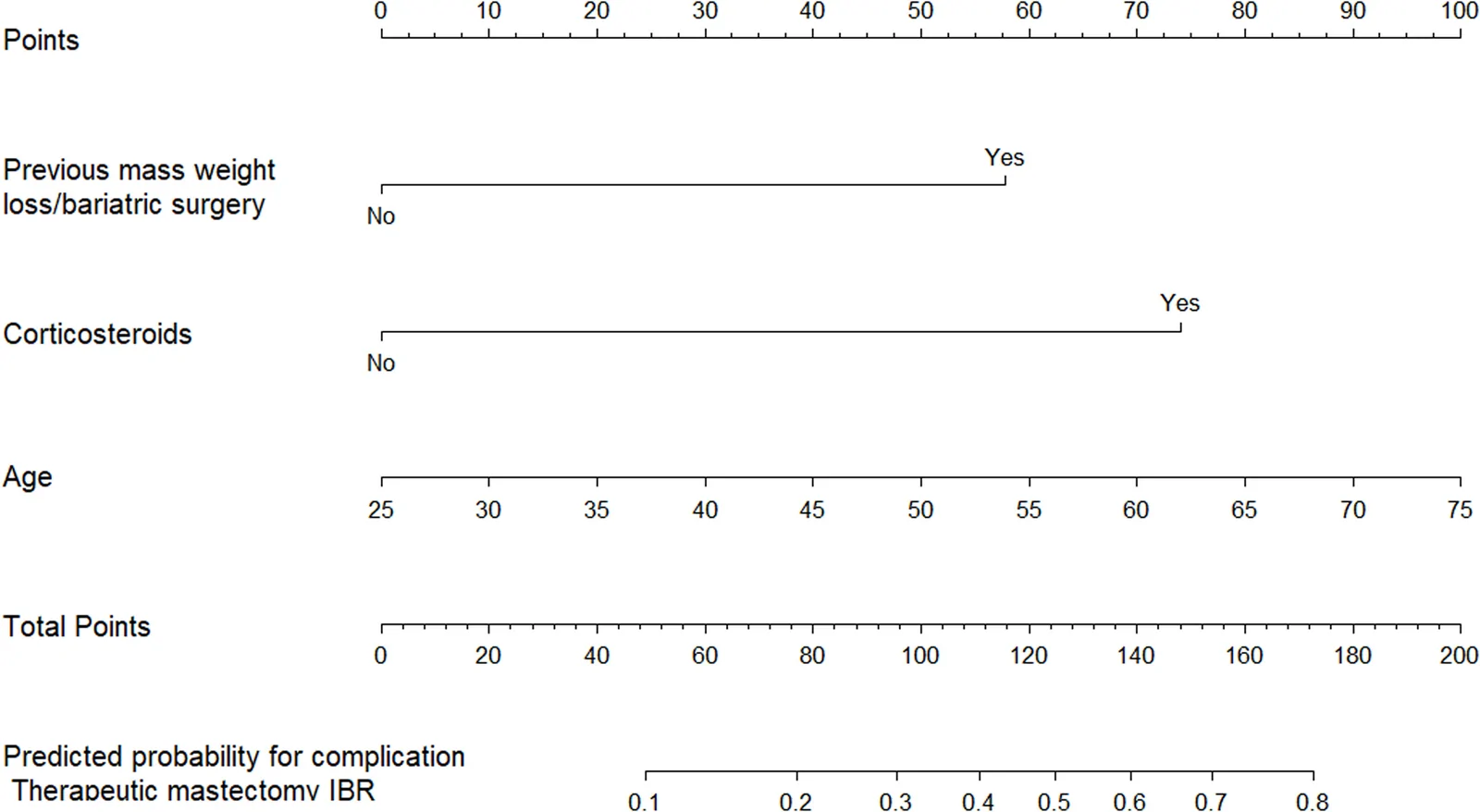
Nomogram for prediction of any complication in the immediate breast reconstruction. Any complication was defined as the occurrence of any of the following ICD-10 codes during the first 90 days after the breast reconstruction: T81.3 (rupture of surgical wound), T81.4 (wound healing complication), T81.0 (hematoma after surgery), HAD 50 (implant loss), A41 (septicemia), I80-I80.9, I 26, I81 (deep vein thrombosis/pulmonary embolism), I21 (myocardial infarction), and I61–64 (stroke).

**Fig. 2:**
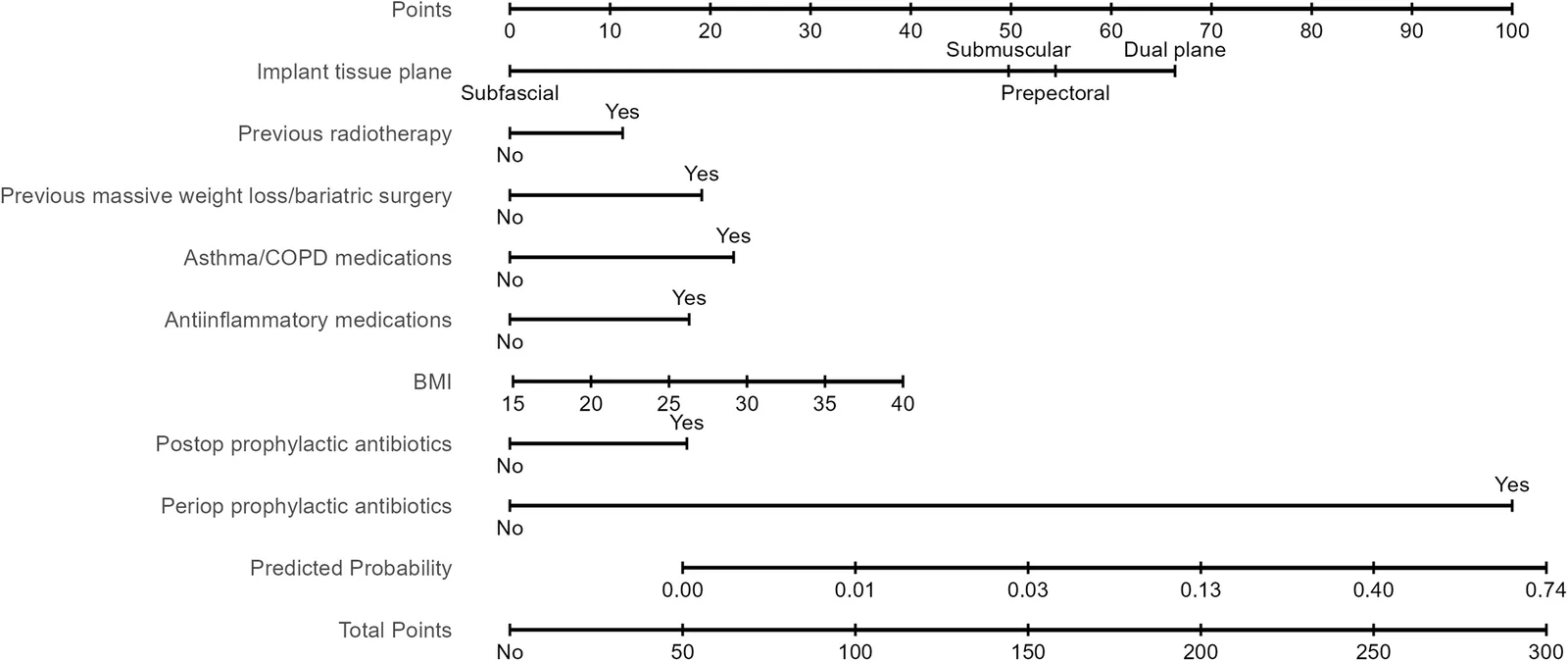
Nomogram for prediction of any complication in delayed breast reconstruction. Any complication was defined as the occurrence of any of the following ICD-10 codes during the first 90 days after the breast reconstruction: T81.3 (rupture of surgical wound), T81.4 (wound healing complication), T81.0 (hematoma after surgery), HAD 50 (implant loss), A41 (septicemia), I80-I80.9, I 26, I81 (deep vein thrombosis/pulmonary embolism), I21 (myocardial infarction), and I61–64 (stroke).

**Fig. 3:**
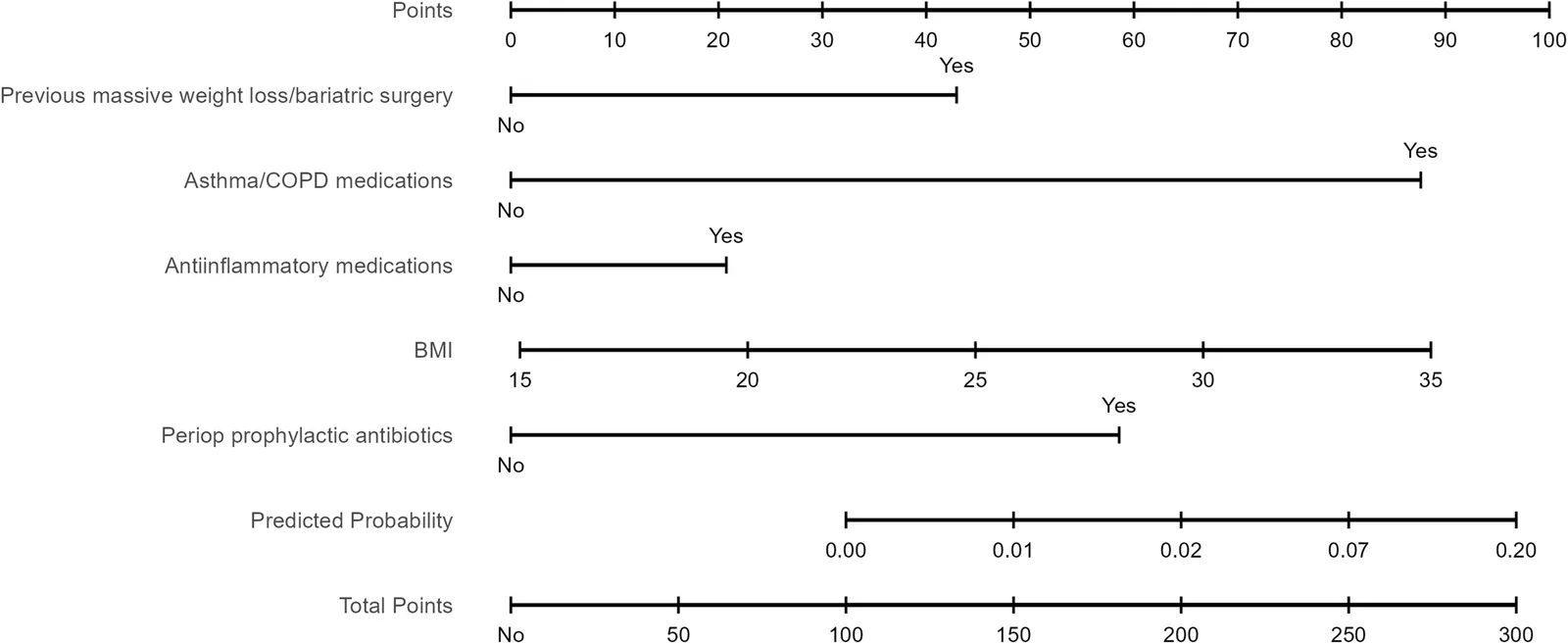
Nomogram for prediction of re-admission in delayed breast reconstruction. Re-admission was defined as any inpatient admission to specialist health care within 90 days after the breast reconstruction, independent of ICD code.

In the bootstrap analysis, a reduced model using the 60% selection threshold showed superior statistical performance, as confirmed by the likelihood ratio test (Table 3, Table 4). Based on these findings, three predictive models were developed. The final predictive model for any complication in the IBR group included previous bariatric operation/MWL, corticosteroids within 3 months of breast reconstruction, and age at breast reconstruction (Fig. 1). In the DBR group, two final predictive models were developed. The model for any complication included previous bariatric operation/MWL, anti-inflammatories within 3 months of breast reconstruction, BMI at breast reconstruction, radiotherapy before breast reconstruction, asthma/chronic obstructive pulmonary disease medication within 3 months of breast reconstruction, tissue plane, and peri‑operative and post-operative antibiotics (Fig. 2). The model for re-admission included bariatric operation/MWL, anti-inflammatories within 3 months of breast reconstruction, BMI at breast reconstruction, asthma/chronic obstructive pulmonary disease medication within 3 months of breast reconstruction, and peri‑operative antibiotics (Fig. 3). The nomograms were constructed using the final models (Fig. 1, Fig. 2, Fig. 3).

**Table 3:** Predictors selected by least absolute shrinkage and selection operator (LASSO) regression for the immediate reconstruction group.

Predictor selected by LASSO	OR (95% CI)	p-value	Frequency of variable inclusion across bootstrap iterations
**Any complication**			
Previous bariatric operation/MWL	5.34 (1.85–15.37)	P = 0.0011	95%
Corticosteroids 3 months before the breast reconstruction	8.55 (2.4–31.6)	P = 0.0037	87%
Age at breast reconstruction	1.06 (1.00–1.12)	p = 0.043,996	68%
Number of tumour operations			66%
Prepectoral placement of implant			46%
Radiotherapy on reconstructed breast			46%
Contralateral surgery at the same time as reconstruction			44%
Invasive cancer			43%
Antiinflammatory medications 3 months before the breast reconstruction			43%
**Re-admission during the first 90 days after the operation**			
Number of tumour operations	16.3 (6.9–41.4)	P < 0.001	100%
Corticosteroids 3 months before the breast reconstruction			45%
Previous bariatric operation/MWL			43%
Asthma/COPD drug 3 months before the breast reconstruction			43%
Age at breast reconstruction			42%

**Table 4:** Predictors selected by least absolute shrinkage and selection operator (LASSO) regression for the delayed reconstruction group.

Predictor selected by LASSO	OR (95% CI)	p-value	Frequency of variable inclusion across bootstrap iterations
**Any complication**			
Perioperative antibiotics	19.0 (2.7–2413.8)	<0.001	100%
Tissue plane Submuscular vs Subfascial Prepectoral vs Subfascial Dual plane vs Subfascial	4.3 (0.5–568.2) 5.0 (0.6–658.7) 7.1 (0.8–938.1)	0.22 0.18 0.09	85%
Postoperative antibiotics	1.7 (1.0–3.0)	0.07	81%
Antiinflammatory medications 3 months before the breast reconstruction	1.7 (0.7–3.6)	0.20	73%
Asthma/COPD medication 3 months before the breast reconstruction	0.5 (0.2–1.8)	0.29	73%
BMI at breast reconstruction	1.03 (1.0–1.1)	0.24	68%
Previous bariatric operation/MWL	1.8 (0.6–4.6)	0.29	63%
Vitamins and minerals 3 months before the breast reconstruction	1.4 (0.7–2.6)	0.30	61%
Previous radiotherapy			60%
Corticosteroids 3 months before the breast reconstruction			60%
Type of implant			59%
Anticoagulantia 3 months before the breast reconstruction			53%
Skin incision			50%
Use of acellular dermal matrix/mesh			49%
Implant texture			49%
Aesthetic re-operation			47%
Age at breast reconstruction			42%
**Re-admission the first 90 days following breast reconstruction**			
Perioperative antibiotics	3.8 (1.0–34.1)	0.06	97%
Implant type			94%
Asthma/COPD medications 3 months before the breast reconstruction	7.3 (0.8–985.6)	0.09	93%
Implant texture			88%
BMI at breast reconstruction	1.1 (1.0–1.2)	0.02	87%
Tissue plane			86%
Anticoagulantia 3 months before the breast reconstruction			85%
Antibiotics intraoperative			83%
Skin incision			81%
Age at breast operation			77%
Corticosteroids 3 months before the breast reconstruction			68%
Antiinflammatory medications 3 months before the breast reconstruction	1.6 (0.6–3.8)	0.33	65%
Aesthetic re-operation			65%
Previous bariatric operation/MWL	2.6 (0.8–7.6)	0.12	62%
Radiotherapy before reconstruction			57%
Vitamins and minerals 3 months before the breast reconstruction			53%
Use of acellular dermal matrix/mesh			52%
Implant shape			48%
Post-operative antibiotics			48%

## Discussion

In this nationwide, population-based study, prediction models for complications following immediate and delayed implant-based breast reconstruction, explicitly incorporating prior massive weight loss (MWL) after bariatric surgery as a candidate predictor, were developed. The findings demonstrate that prior bariatric surgery/MWL is an independent and consistently selected risk factor for postoperative complications in both immediate and delayed breast reconstruction re-admission in delayed reconstruction. To our knowledge, this is the first study to quantify the contribution of MWL to cumulative complication risk in breast reconstruction and to integrate it into clinically applicable prediction models.

These findings support the hypothesis that MWL contributes to an increased cumulative risk of complications. The inclusion of MWL as a predictor in all final models underscores its relevance as a risk factor in implant-based breast reconstruction, alongside previously established risk factors such as BMI, radiotherapy, comorbidity, and surgical techniques^1^^,^^2^^,^^4^, ^5^, ^6^, ^7^, ^8^, ^9^ The association between MWL and complications has previously been seen for other types of plastic surgical operations^14^^–^.^17^ The observed increased complication risk among MWL patients is consistent with findings from body contouring surgery, where post-bariatric patients have been shown to experience significantly higher rates of wound complications and re-operations compared with non-MWL patients.^18^ The results suggest that similar risk patterns apply to reconstructive breast surgery. In addition, nutritional deficiencies following bariatric surgery, such as protein, iron, zinc, and vitamin deficiencies, may further impair tissue repair and immune function.^15^, ^16^, ^17^ These factors may be particularly consequential in breast reconstruction, where tissue viability and wound healing are critical determinants of outcome.

A few surprising risk factors were selected in the models and warrant comment. Notably, the use of antibiotics perioperatively and postoperatively in the DBR cohort. The predictors contributed significantly to the models, even though antibiotics themselves cannot cause the type of complications analyzed in the study. Antibiotic use was included in the analysis, with the hypothesis that non-use of antibiotics might increase the risk of complications. However, the finding that antibiotic use is an independent risk factor for complications is clinically unlikely, and it is therefore interpreted as a possible confounder, reflecting other unknown risk factors, that might increase the tendency to prescribe antibiotics and that we have not included in the analysis. No prediction model could be created for the risk of re-admission in the IBR group, as only one predictor was selected. In addition, the only selected predictor, several tumour operations due to non-radical tumour excision (Table 3), is probably an anomaly, as the operations themselves are likely to have led to re-admission, as registered in PAR, and this might explain the exceptionally high odds ratio of 16.3.

The major strengths of this study include its nationwide, population-based design; the use of high-quality, prospectively collected registry data; and a robust statistical approach that incorporates group LASSO and bootstrap resampling to enhance model stability. However, several limitations should be acknowledged. First, due to limitations in the data from the national registries used, a few important previously known predictors could not be analyzed and incorporated into the current models. Notably, smoking in all three models and BMI in the IBR model. These variables could have affected the model's risk estimates, reduced its certainty, and prevented it from accurately predicting the true cumulative risk for patients. Moreover, it is difficult to study a high BMI in a Swedish population, as only women with a BMI under 30 are eligible for breast reconstruction. It is theoretically possible that there were more smokers than non-smokers who had a higher BMI, more bariatric surgery/MWL and radiation in their history, took more corticosteroids, anti-inflammatory medications, asthma/chronic obstructive pulmonary disease medication, and antibiotics, were older at breast reconstruction within 3 months, and were operated on with different surgical techniques. Hence, it is not possible to exclude that the lack of smoking status affected the predictors of the models. In addition, it was not possible to investigate the effect of reconstruction timing (immediate vs. delayed) because different data were available for the two groups, and they therefore had to be analysed separately. Other variables for which some data were insufficient. Moreover, data were insufficient/missing regarding surgical techniques details, such as prepectoral placement, implant volume, and usage of temporary tissue expanders (TEs). The proportion of permanent implants performed as a single-stage procedure versus a two-stage procedure involving temporary TE remains unclear, since temporary TEs are not recorded in BRIMP. Secondly, the definitions of variables could have affected the results. MWL was defined as a patient who had undergone bariatric surgery according to SOReg. This definition also implies that patients with limited effects of the bariatric operation were included in the study. However, the requirement that women have a BMI under 30 to be eligible for breast reconstruction in combination with a mean pre-bariatric BMI of 39 (4.1) (Table 2) means that most of the women had lost a substantial amount of weight. The definition of MWL also implies that there could have been patients with MWL due to other causes, such as medically induced MWL, among the controls. However, the majority of included controls were operated before Glucagon-Like peptide-1 (GLP-1) receptor agonists became widely used, so they should not constitute a major confounder. Thirdly, complications were identified through ICD-10 codes in specialist care, which may underestimate minor complications managed in primary care. Similarly, the re-admission rates might be overestimated, as they included admissions for any reason and could have been unrelated to the surgery. Comorbidity was defined as dispensed drug prescriptions during the three months leading up to the breast reconstruction. Although PDR captures all dispensed prescriptions, regardless of who prescribed them, it does not provide information about the severity of individual patients’ comorbidities. In the case of preoperative antibiotics, this might be a marker of a higher risk of complications rather than a risk factor itself. Moreover, the number of individuals with certain drugs and comorbidities was relatively small, making the analysis less robust. Finally, validation in independent cohorts is required before widespread clinical implementation of the nomograms.

Future research should aim to externally validate the proposed prediction models and explore whether incorporating smoking and BMI affects them. In addition, better data are needed to draw conclusions about the effects of specific surgical techniques. More studies are needed to determine how MWL achieved through pharmacological or lifestyle interventions without surgery affects risk. Regarding post-bariatric MWL, further studies are needed to develop population-specific risk prediction models and to determine whether incorporating more detailed measures, such as nutritional markers, sarcopenia, and the magnitude and timing of weight loss, can further improve risk prediction. In addition, studies comparing different reconstructive strategies in MWL patients may help identify approaches that mitigate complication risk in this growing patient population.

From a clinical perspective, the findings suggest that MWL should be explicitly considered during preoperative risk assessment. Patients with prior bariatric surgery may benefit from targeted optimisation strategies, including nutritional assessment and supplementation, careful selection of reconstructive technique, and heightened postoperative surveillance. Furthermore, the results indicate that there is a need for nuanced risk–benefit discussions, for example, when bariatric surgery is used as a bridge therapy in patients with obesity seeking breast reconstruction,^13^ regarding how long time should elapse between the MWL and the reconstruction.

In conclusion, this nationwide study demonstrates that previous post-bariatric massive weight loss might be an independent and clinically meaningful risk factor for complications after both immediate and delayed breast reconstruction. Incorporating MWL into cumulative risk prediction models improves individualised risk assessment and supports shared decision-making. As the number of patients with MWL requiring breast reconstruction continues to rise, recognising MWL as a risk factor is essential for optimising outcomes and patient care.

## Declarations

### Ethics approval and consent to participate

The study was vetted and approved by the Swedish Ethical Review Authority (2024–01,494-01). It followed the principles of the Helsinki Declaration, as coded in Swedish law, the "Ethics Review Act” SFS 2003:460. Data were processed in accordance with the General Data Protection Regulation (GDPR) and the study's data management plan. Informed consent is waived for national registry-based studies in Sweden, as the data are pseudonymized. Furthermore, before patients are enrolled, they are given the possibility to opt out.

### Consent for publication

Informed consent is waived for national registry-based studies in Sweden, as the data are pseudonymized. Furthermore, before patients are enrolled, they are given the possibility to opt out.

### Data of availability

The patients and controls of this study did not give written consent for their data to be shared publicly, so due to the sensitive nature of the research, supporting data is not available.

## Funding

The study was funded by grants from the Swedish Cancer Society [21 0279 SCIA] and the federal government under the ALF agreement [ALFGBG-1005,048]. The sources of funding had no role in the design of the study, data collection, analysis, interpretation, or writing of the manuscript. Gothenburg University, the BIBSAM agreement, funded open access publication.

## Authors' contributions

EH: Conceptualisation, methodology, writing—original draft preparation, visualisation, funding acquisition. YL: methodology, formal analysis, writing—review and editing, AG-E: methodology, formal analysis, writing—review and editing, AP: Conceptualisation, methodology, data curation, visualisation, writing—review and editing

## Sources of funding

The study was funded by grants from the Swedish Cancer Society [21 0279 SCIA] and the federal government under the ALF agreement [ALFGBG-1005,048]. The funding sources had no role in the design of the study, data collection, analysis, interpretation, or the writing of the manuscript. Open access publication was funded by Gothenburg University through the BIBSAM agreement.

**Category:** Original article, observational study

**The data have never previously been reported or presented**

## Declaration of competing interest

The authors declare that they have no competing interests and no topically related relationships. EH has funding as reported under the sources of funding section. EH is part of the steering committee for the Swedish Breast Implant registry.

## Appendix Supplementary materials

Image, application 1
