# Supplementary material for: Risk prediction of 90-day complications in implant-based breast reconstruction –does previous post-bariatric massive weight loss add to the risk? A population-based study
Source: JPRAS Open. 2026 Aug 8;52:15–26. doi: 10.1016/j.jpra.2026.07.041 (PMC13543791; doi:10.1016/j.jpra.2026.07.041)
Supplement: Supplementary file 1 [file mmc1.docx]

# Supplementary tables

## Supplementary Table 1. Previous studies of prediction models stating odds ratios for the risk of complications in breast reconstruction

| **Risk factor** | | **Blok, 2024, the Netherlands [1]** | **Kooijman, 2025, the Netherlands [2]** | **Naoum, 2021, USA [3]** | | **Hassan, 2023, USA [4]** | | | | | **Palve, 2020, Finland [5]** |
| --- | --- | --- | --- | --- | --- | --- | --- | --- | --- | --- | --- |
| *Study design* | | *Population-based study based on the Dutch implant registry* | *Single-centre consecutive cohort* | *Single-centre consecutive cohort* | | *Single-centre consecutive cohort* | | | | | *Single-centre consecutive cohort* |
| *Number of reconstructions/patients* | | *NR/5260* | *1989/1512* | *(1817/1817)*  *1235/1235 implant/TE-based* | | *481/694* | | | | | *(850/793)*  *51 implant-based reconstructions and 151 LD+implant* |
| *Timing of reconstruction* | | *Immediate* | *Immediate* | *Immediate* | | *Immediate* | | | | | *Immediate and delayed* |
| *Type of reconstruction* | | *Implant-based* | *Implant-based* | *All types* | | *Two-staged implant-based* | | | | | *All types* |
| *Complication investigated* | | *Implant loss within 6 months* | *Complications requiring surgical re-intervention or implant loss within 16 weeks* | *Capsular contracture requiring capsulotomy* | *Implant loss* | *Implant infection* | | | *Implant loss* | | *Any complication* |
| **BMI** OR (95% CI), p-value | | 1.050 (1.023-1077), p<0.001 | NS | NS |  | 1.07 (1.03-1.12), p<0.001 | | | OR 1.07; 95% CI 1.02-1.13, p=0.004 | | 1.59 (1.05–2.39), p = 0.027 |
| **Active smoking** OR (95% CI), p-value | | 2.081 (1.513-2.862) p<0.001 | 2.72 (1.97–3.76), p<0.001 |  | NS |  | | |  | | NS |
| **Comorbidities**  OR (95% CI), p-value | **Comorbidity NOS** |  | 1.43 (1.10–1.88), p=0.009 |  |  |  | | |  | |  |
|  | **Diabetes** |  |  |  |  |  |  |  |  |  | NS |
|  | **Hypertension** |  |  |  |  |  |  |  |  |  | NS |
|  | **COPD** |  |  |  |  |  |  |  |  |  | 2.77 (1.50–5.12), p = 0.001 |
| **Ethnicity** OR (95% CI), p-value | |  |  |  | 2.57 (1.18–5.58) p=0.02 |  | | |  | |  |
| **Age** OR (95% CI), p-value | |  | NS |  |  | 1.03 (1.01-1.05), p=0.003 | | |  | | NS |
| **Radiotherapy**  OR (95% CI), p-value | Previous radiotherapy | 1.881 (1.06-3.081), p=0.029 |  |  |  | | 2.31 (1.00-5.31), p=0.049 | NS | | NS | |
|  | Radiotherapy without boost |  |  | 2.11 (1.00–4.43) p=0.05 | NS | |  |  | |  |  |
|  | Radiotherapy with boost |  |  | 2.52 (1.20–5.25) p=0.01 | 5.71 (2.83-11.5) p<0.0001 | |  |  | |  |  |
|  | Proton radiotherapy |  |  | 15.3 (6.1–38.5), p <0.0001 | NS | |  |  | |  |  |
|  | Radiotherapy NOS |  |  |  |  | |  |  | |  |  |
| **Neoadjuvant chemotherapy** OR (95% CI), p-value | |  |  | NS |  | |  | OR 2.07; 95% CI 1.22-3.53, p=0.007 | |  | |
| **Prepectoral placement of implant** OR (95% CI), p-value | | 1.911 (1.346-2.713), p<0.001 |  |  |  | | NS | NS | |  | |
| **Use of acellular dermal matrix (ADM)/mesh** OR (95% CI), p-value | |  |  |  | 3.82 (1.24–11.8) p=0.02 (Flex HD mesh) | |  |  | |  | |
| **Mastectomy weight**  OR (95% CI), p-value | |  | 1.77 (1.29–2.43) p<0.001  (>425 gr) |  |  | |  |  | |  | |
| **Implant volume**  OR (95% CI), p-value | | 1.306 (1.109-1.539), p=0.001 | 1.44 (1.06–1.96), p=0.020  (>422 gr) |  |  | |  |  | |  | |
| **Duration of operation**  OR (95% CI), p-value | |  | 1.27 (1.01–1.60), p=0.039  (>143 gr) |  |  | |  |  | |  | |
| **Experience of surgeon**  OR (95% CI), p-value | |  | 1.82 (I1.38–2.39), p<0.001  (Mastectomy was performed by a non-staff oncologic surgeon) |  |  | |  |  | |  | |
| **Latissimus dorsi flap compared to just implant**  OR (95% CI), p-value | |  |  |  |  | |  |  | | 4.05 (2.10–7.81), p < 0.001 | |
| **Timing – immediate compared to delayed**  OR (95% CI), p-value | |  |  |  |  | |  |  | | 0.69 (0.44–1.07), p = 0.099 | |

Grey = not studied/analysed

CI – confidence interval

COPD – chronic obstructive pulmonary disease

LD - Latissimus dorsi

LOS – Length of stay

NOS – not otherwise specified

NS – statistically non-significant

OR – odds ratio

S-significant

TE -tissue expander

1.	Blok YL, Plat VD, van der Hage JA, Putter H, Krekel NMA, Mureau MAM. Risk Prediction of Implant Loss following Implant-Based Breast Reconstruction: A Population-Based Study. Plast Reconstr Surg. 2025;156(1):1e-10e. Epub 20241217.

2.	Kooijman MML, Woerdeman LAE, van Duijnhoven FH, Scholten AN, van Bommel ACM, Breugem CC. Short-term complications of immediate implant-based breast reconstruction: Risk factor analyses and a novel cumulative risk algorithm. J Plast Reconstr Aesthet Surg. 2025;111:134-44. Epub 20250912.

3.	Naoum GE, Ho AY, Shui A, Salama L, Goldberg S, Arafat W, et al. Risk of Developing Breast Reconstruction Complications: A Machine-Learning Nomogram for Individualized Risk Estimation with and without Postmastectomy Radiation Therapy. Plast Reconstr Surg. 2022;149(1):1e-12e.

4.	Hassan AM, Biaggi-Ondina A, Asaad M, Morris N, Liu J, Selber JC, et al. Artificial Intelligence Modeling to Predict Periprosthetic Infection and Explantation following Implant-Based Reconstruction. Plast Reconstr Surg. 2023;152(5):929-38. Epub 20230303.

5.	Palve JS, Luukkaala TH, Kaariainen MT. Predictive risk factors of complications in different breast reconstruction methods. Breast Cancer Res Treat. 2020;182(2):345-54. Epub 20200528.

## Supplementary Table 2. Candidate predictors in patients who were and were not readmitted after the breast reconstruction, and differences between the groups

| **Candidate predictors for any complication** | **Immediate breast reconstruction** | | | **Delayed breast reconstruction** | | |
| --- | --- | --- | --- | --- | --- | --- |
|  | **Patients who had no complication**  N=179*^1^* | **Patients who had a complication**  N=66*^1^* | **p-value***^2^* | **Patients who had no revision**  N=550*^1^* | **Patients who had a complication**  N=52*^1^* | **p-value***^2^* |
| **Characteristics of participants** | | | | | | |
| Postbariatric/MWL |  |  | 0.4 |  |  | 0.12 |
| No | 161 (90%) | 57 (86%) |  | 519 (94%) | 46 (88%) |  |
| Yes | 18 (10%) | 9 (14%) |  | 31 (5.6%) | 6 (12%) |  |
| Missing | 0 | 0 |  | 0 | 0 |  |
| BMI at breast reconstruction | Could not be analysed due to missing data | |  | 25.5 (23.6, 27.8) | 27.3 (25.1, 29.6) | **0.004** |
| Missing |  |  |  | 7 | 4 |  |
| Percentage of BMI lost | Could not be analysed due to missing data | |  | Could not be analysed due to missing data | |  |
| Age at breast reconstruction (years) | 48 (42, 53) | 46 (42, 54) | 0.8 | 52 (44, 60) | 51 (47, 58) | 0.5 |
| Missing |  |  |  | 4 | 2 |  |
| Smoking | Could not be analysed due to missing data | |  |  | |  |
| Post-operative complications the first 6 weeks after the bariatric operation | Could not be analysed due to missing data | |  |  | | >0.9 |
| No |  |  |  | 548 (100%) | 52 (100%) |  |
| Yes |  |  |  | 2 (0.4%) | 0 (0%) |  |
| **Comorbidity expressed as medication (ACT-codes) taken 3 months before the breast reconstruction** | | | | | | |
| Diabetes medication (A10) |  |  | 0.6 |  |  | 0.6 |
| No | 175 (98%) | 66 (100%) |  | 537 (98%) | 52 (100%) |  |
| Yes | 4 (2.2%) | 0 (0%) |  | 13 (2.4%) | 0 (0%) |  |
| Vitamins and minerals (A11-A12) |  |  | 0.6 |  |  | 0.9 |
| No | 164 (92%) | 59 (89%) |  | 483 (88%) | 46 (88%) |  |
| Yes | 15 (8.4%) | 7 (11%) |  | 67 (12%) | 6 (12%) |  |
| Cardiac drugs (C01) |  |  | 0.5 |  |  | 0.2 |
| No | 178 (99%) | 65 (98%) |  | 549 (100%) | 51 (98%) |  |
| Yes | 1 (0.6%) | 1 (1.5%) |  | 1 (0.2%) | 1 (1.9%) |  |
| Anticoagulants (B01) |  |  | 0.7 |  |  | 0.060 |
| No | 172 (96%) | 63 (95%) |  | 509 (93%) | 44 (85%) |  |
| Yes | 7 (3.9%) | 3 (4.5%) |  | 41 (7.5%) | 8 (15%) |  |
| Corticosteroids (H02) |  |  | 0.2 |  |  | 0.055 |
| No | 157 (88%) | 54 (82%) |  | 510 (93%) | 44 (85%) |  |
| Yes | 22 (12%) | 12 (18%) |  | 40 (7.3%) | 8 (15%) |  |
| Anti-inflammatory and antirheumatic drugs (M01) |  |  | 0.8 |  |  | 0.4 |
| No | 168 (94%) | 63 (95%) |  | 503 (91%) | 46 (88%) |  |
| Yes | 11 (6.1%) | 3 (4.5%) |  | 47 (8.5%) | 6 (12%) |  |
| Neuroleptics, sedatives and sleeping drugs (N05) |  |  | >0.9 |  |  | >0.9 |
| No | 162 (91%) | 60 (91%) |  | 547 (99%) | 52 (100%) |  |
| Yes | 17 (9.5%) | 6 (9.1%) |  | 3 (0.5%) | 0 (0%) |  |
| Asthma/COPD drug (R03) |  |  | 0.3 |  |  | 0.2 |
| No | 170 (95%) | 65 (98%) |  | 521 (95%) | 52 (100%) |  |
| Yes | 9 (5.0%) | 1 (1.5%) |  | 29 (5.3%) | 0 (0%) |  |
| Blood clotting drugs (B02) |  |  | >0.9 |  |  | **0.010** |
| No | 178 (99%) | 66 (100%) |  | 547 (99%) | 49 (94%) |  |
| Yes | 1 (0.6%) | 0 (0%) |  | 3 (0.5%) | 3 (5.8%) |  |
| **Oncological factors** | | | | | | |
| Invasive cancer |  |  | 0.6 | Could not be analysed due to missing data | |  |
| No | 107 (65%) | 42 (69%) |  |  |  |  |
| Yes | 57 (35%) | 19 (31%) |  |  |  |  |
| Missing | 15 | 5 |  |  |  |  |
| Neoadjuvant chemotherapy |  |  | >0.9 | NA | |  |
| No | 153 (85%) | 56 (85%) |  |  |  |  |
| Yes | 26 (15%) | 10 (15%) |  |  |  |  |
| Radiotherapy after the reconstruction |  |  | 0.5 | NA | |  |
| No | 80 (58%) | 31 (63%) |  |  |  |  |
| Yes | 59 (42%) | 18 (37%) |  |  |  |  |
| Missing | 40 | 17 |  |  |  |  |
| Radiotherapy before the reconstruction |  | |  |  |  | >0.9 |
| No |  |  |  | 408 (80%) | 40 (80%) |  |
| Yes |  |  |  | 103 (20%) | 10 (20%) |  |
| Missing |  |  |  |  |  |  |
| Number of tumour operations |  |  | **<0.001** | NA | |  |
| 0 | 163 (91%) | 27 (41%) |  |  |  |  |
| 1 | 12 (6.7%) | 31 (47%) |  |  |  |  |
| >=2 | 4 (2.2%) | 8 (12%) |  |  |  |  |
| >=2 |  |  |  |  |  |  |
| Axillary surgery |  |  | >0.9 | Not available in the registry | |  |
| No | 12 (6.7%) | 4 (6.1%) |  |  |  |  |
| Yes | 167 (93%) | 62 (94%) |  |  |  |  |
| **Factors related to breast reconstruction** | | | | | | |
| Contralateral surgery at the same time as the reconstruction |  |  | >0.9 | Not available in the registry | |  |
| No | 157 (88%) | 58 (88%) |  |  |  |  |
| Yes | 22 (12%) | 8 (12%) |  |  |  |  |
| Usage of acellular dermal matrix or mesh |  |  | 0.2 |  |  | 0.2 |
| No | 133 (74%) | 54 (82%) |  | 534 (97%) | 49 (94%) |  |
| Yes | 46 (26%) | 12 (18%) |  | 16 (2.9%) | 3 (5.8%) |  |
| Prepectoral placement of implant |  |  | 0.8 | Classified in another way in the registry | |  |
| No | 150 (84%) | 56 (85%) |  |  |  |  |
| Yes | 29 (16%) | 10 (15%) |  |  |  |  |
| Tissue plane | Classified in another way in the registry | |  |  |  | 0.075 |
| Submuscular |  |  |  | 331 (64%) | 27 (56%) |  |
| Pre-pectoral |  |  |  | 90 (18%) | 7 (15%) |  |
| Subfascial |  |  |  | 7 (1.4%) | 3 (6.3%) |  |
| Dual plane |  |  |  | 86 (17%) | 11 (23%) |  |
| Missing |  |  |  | 36 | 4 |  |
| Type of implant | Could not be analysed due to missing data | |  |  |  | 0.080 |
| Implant |  |  |  | 321 (59%) | 23 (46%) |  |
| Permanent tissue expander |  |  |  | 225 (41%) | 27 (54%) |  |
| Missing |  |  |  | 4 | 2 |  |
| Implant shape | Data not available in the registry | |  |  |  | 0.5 |
| Round^4^ |  |  |  | 66 (12%) | 8 (15%) |  |
| Anatomical |  |  |  | 484 (88%) | 44 (85%) |  |
| Missing |  |  |  |  |  |  |
| Implant surface | Data not available in the registry | |  |  |  | 0.6 |
| Smooth/nano textured |  |  |  | 14 (2.5%) | 0 (0%) |  |
| Micro/mactro textured |  |  |  | 536 (97%) | 52 (100%) |  |
| Polyurethan |  |  |  | 0 | 0 |  |
| Implant volume | Could not be analysed due to missing data | |  | Could not be analysed due to missing data | |  |
| Incision | Data not available in the registry | |  |  |  | 0.12 |
| Mastectomy scar |  |  |  | 327 (66%) | 25 (54%) |  |
| Other |  |  |  | 169 (34%) | 21 (46%) |  |
| Missing |  |  |  | 54 | 6 |  |
| Lipofilling | Data not available in the registry | |  |  |  | 0.2 |
| Yes |  |  |  | 542 (99%) | 50 (96%) |  |
| No |  |  |  | 8 (1.5%) | 2 (3.8%) |  |
| Aesthetic re-operation | Data not available in the registry | |  |  |  | 0.7 |
| No |  |  |  | 476 (87%) | 46 (88%) |  |
| Yes |  |  |  | 74 (13%) | 6 (12%) |  |
| Perioperative antibiotics (at induction) | Could not be analysed due to missing data | |  |  |  | **0.031** |
| No |  |  |  | 63 (12%) | 1 (2.1%) |  |
| Yes |  |  |  | 442 (88%) | 47 (98%) |  |
| Missing |  |  |  | 45 | 4 |  |
| Intraoperative antibiotics (rinse) | Data not available in the registry | |  |  |  | 0.11 |
| No |  |  |  | 471 (98%) | 43 (93%) |  |
| Yes |  |  |  | 11 (2.3%) | 3 (6.5%) |  |
| Missing |  |  |  | 68 | 6 |  |
| Postoperative antibiotics | Data not available in the registry | |  |  |  | >0.9 |
| No |  |  |  | 268 (54%) | 25 (54%) |  |
| Yes |  |  |  | 225 (46%) | 21 (46%) |  |
| Missing |  |  |  |  |  |  |

^1^Median (Q1, Q3); n (%)

^2^Wilcoxon rank sum test; Fisher's exact test; NA; Pearson's Chi-squared test

^3^Variables are defined in the method section

^4^ Ergonomic implants are registered as ‘round’ in BRIMP (<https://registercentrum.blob.core.windows.net/brimp/r/Br-stimplantatregistret-Prim-roperation-2024-05-17-tvALbnphv.pdf>)

BMI Body mass index

MWL massive weight loss

## Supplementary Table 3. Complications in the different groups

| **Complications** | **Immediate breast reconstruction** | **Delayed breast reconstruction** |
| --- | --- | --- |
| Number of patients | 243 | 498 |
| Number of reconstructed breasts | 245 | 602 |
| Number of breasts with complications | 40 (16.3%) | 87 (14.5%) |
| Systemic complications^1^ (per patient) | 2 (0.8%) | 9 (1.5%) |
| *Pulmonary complications* | *0* | *1* |
| *Pulmonary embolism or deep vein thrombosis* | *1 (2^2^)* | *8* |
| *Septicemia* | *1* | *0* |
| Breast related complications^3^ (per breast) | 38 (15.5%) | 81 (13.5%) |
| *Wound healing complication/infection, excluding implant loss* | *30 (12.2%)* | *66 (11.0%)* |
| *Implant loss* | *8 (3.3%)* | *15 (2.5%)* |
| Re-admission 90 days^4^ (per patient) | 66 (26.9%) | 44 (8.8%) |

1. ICD-codes I21, I61-64, I80, I81, I26, J18.0-9, J69.0, J80, J98.1, A41
2. The patient had bilateral deep vein thromboses
3. ICD-codes T81.3-4, T81.0, T85.7, T85.4, HAD50
4. Re-admission independent of ICD codes. Thus, the re-admissions included admissions with ICD codes other than the codes specified for breast-related complications in the study.
